# Supplementary material for: A Novel Magnetic Resonance Imaging-Based Radiomics and Clinical Predictive Model for the Regrowth of Postoperative Residual Tumor in Non-Functioning Pituitary Neuroendocrine Tumor
Source: Medicina (Kaunas). 2023 Aug 23;59(9):1525. doi: 10.3390/medicina59091525 (PMC10535289; doi:10.3390/medicina59091525)
Supplement: Supplementary file 1 [file medicina-59-01525-s001.zip › Supplementary File/Table S1.docx]

**Table S1.** Baseline characteristics of patients (N = 114).

| **Variables (N = 114)** | **Training set (N = 80)** | **Test set (N = 34)** | **Statistics P** |
| --- | --- | --- | --- |
|  |  |  |  |
| **Gender, n (%)**  Male  Female | 46 (57.5%)  34 (42.5%) | 21 (61.8%)  13 (38.2%) | 0.672 |
| **Age (years)** | 53.5 (41.5-66.0) | 49.0 (43.0-66.0) | 0.867 |
| **Weight (kg)** | 64.0 (58.5-70.0) | 60.5 (54.8-66.2) | 0.083 |
| **Height (m)** | 1.64±0.06 | 1.64±0.07 | 0.775 |
| **BMI** | 23.85 (21.26-26.42) | 22.12 (21.00-24.80) | 0.077 |
| **Headache, n (%)**  Yes  No | 38 (47.5%)  42 (52.5%) | 16 (46.2%)  18 (53.8%) | 0.966 |
| **Vision changes, n (%)**  Yes  No | 43 (53.8%)  37 (46.2%) | 22 (64.7%)  12 (35.3%) | 0.280 |
| **Pituitary apoplexy,**  **n (%)**  Yes  No | 10 (12.5%)  70 (87.5%) | 4 (11.8%)  30 (88.2%) | 0.913 |
| **Knosp grade, n (%)**  0  1  2  3  4 | 14 (17.5%)  14 (17.5%)  16 (20.0%)  15 (18.8%)  21 (26.2%) | 8 (23.5%)  5 (14.7%)  4 (11.8%)  6 (17.6%)  11 (32.4%) | 0.770 |
| **Cystic, n (%)**  Yes  No | 22 (27.5%)  58 (72.5%) | 9 (26.5%)  25 (73.5%) | 0.910 |
| **Hardy grade, n (%)**  0  I  II  III  IV | 22 (27.5%)  33 (41.2%)  19 (23.8%)  2 (2.5%)  4 (5.0%) | 5 (14.7%)  12 (35.3%)  10 (29.4%)  4 (11.8%)  3 (8.8%) | 0.148 |
| **TVDT, n (%)**  <1 year  ≥1 year | 33 (41.2%)  47 (58.8%) | 16 (47.1%)  18 (52.9%) | 0.567 |
| **Surgical resection ratio**  **(%)** | 68.75 (16.25-98.60) | 75.51 (22.54-98.01) | 0.386 |
| **Consistency, n (%)**  Soft  Medium  Hard | 49 (61.3%)  7 (8.7%)  24 (30.0%) | 25 (73.5%)  2 (5.9%)  7 (20.6%) | 0.454 |
| **Residual position, n (%)**  Intrasellar  Suprasellar  Both | 35 (43.8%)  28 (35.0%)  17 (21.2%) | 15 (44.1%)  13 (38.2%)  6 (17.7%) | 0.894 |
| **Postoperative T1**  **enhancement, n (%)**  Yes  No | 62 (77.5%)  18 (22.5%) | 27 (79.4%)  7 (20.6%) | 0.821 |
| **Postoperative abnormal hormone level, n (%)**  None  Cortisol  Thyroid  Both | 36 (45.0%)  24 (30.0%)  7 (8.8%)  13 (16.2%) | 14 (41.2%)  10 (29.4%)  3 (8.8%)  7 (20.6%) | 0.953 |
| **Postoperative diabetes**  **insipidus, n (%)**  Yes  No | 33 (41.2%)  47 (58.8%) | 12 (35.3%)  22 (64.7%) | 0.552 |
| **Preoperative electrolyte**  Na (mmol/L)  K (mmol/L)  Ca (mmol/L)  P (mmol/L) | 140 (138-142)  3.97 (3.74-4.18)  2.34 (2.24-2.49)  1.95 (1.40-2.41) | 139 (137--141)  3.81 (3.61-4.07)  2.26 (2.17-2.39)  1.57 (1.21-2.71) | 0.341  0.198  0.052  0.460 |
| **Ki-67, n (%)**  <3%  ≥3% | 54 (67.5%)  26 (32.5%) | 27 (79.4%)  7 (20.6%) | 0.200 |

Abbreviation: TVDT, Tumor volume doubling time;
