# Supplementary material for: A Novel Magnetic Resonance Imaging-Based Radiomics and Clinical Predictive Model for the Regrowth of Postoperative Residual Tumor in Non-Functioning Pituitary Neuroendocrine Tumor
Source: Medicina (Kaunas). 2023 Aug 23;59(9):1525. doi: 10.3390/medicina59091525 (PMC10535289; doi:10.3390/medicina59091525)
Supplement: Supplementary file 1 [file medicina-59-01525-s001.zip › Supplementary File/Table S2.docx]

**Table S2.** List of selected radiomics features from mRMR and LASSO regression.

| **Image category** | **Feature name** |
| --- | --- |
| Preoperative |  |
| T1WI | Original_glcm_MCC |
|  | Original_shape_Sphericity |
|  | Wavelet.LHL_glrlm_Run Variance |
| T1CE | Wavelet.HHL_firstorder_Skewness |
|  | Wavelet.LLL_firstorder_Skewness |
|  | Original_shape_Sphericity |
| T2WI | Wavelet.HLL_firstorder_Mean |
|  | Original_shape_Sphericity |
| Postoperative |  |
| T1WI | Wavelet.LLL_glrlm_Long Run High Gray Level Emphasis |
|  | Wavelet.LLH_glszm_Large Area Low Gray Level Emphasis |
|  | Wavelet.HHL_glszm_Large Area High Gray Level Emphasis |
|  | Wavelet.HHL_glcm Cluster Shade |
| T1CE | Original_shape_Sphericity |
|  | Original_shape_Flatness |
| T2WI | Wavelet.HHL_glszm_Small Area Emphasis |
|  | Wavelet.LHH_firstorder Kurtosis |

Abbreviation: T1WI, T1-weighted image; T2WI, T2-weighted image; T1CE, contrast-enhanced;
