# Supplementary material for: A Novel Magnetic Resonance Imaging-Based Radiomics and Clinical Predictive Model for the Regrowth of Postoperative Residual Tumor in Non-Functioning Pituitary Neuroendocrine Tumor
Source: Medicina (Kaunas). 2023 Aug 23;59(9):1525. doi: 10.3390/medicina59091525 (PMC10535289; doi:10.3390/medicina59091525)
Supplement: Supplementary file 1 [file medicina-59-01525-s001.zip › Supplementary File/figue legend.docx]

**Figure S1.** Comparison of efficiency of radiomics features between pre- and postoperative on MRI images. The ROC curve and AUC of radiomics features on T1WI in training **(a)** and test sets **(b)**; The ROC curve and AUC of radiomics features on T2WI in training **(c)** and test sets **(d)**; The ROC curve and AUC of radiomics features on T1CE in training **(e)** and test sets **(f)**; Pre, preoperative radiomics features. Post, postoperative radiomics features. Pre+Post, the combination of pre- and postoperative radiomics features.

**Table S1.** Baseline characteristics of patients (N = 114).

**Table S2.** List of selected radiomics features from mRMR and LASSO regression.
